# TRAP1 induced cisplatin resistance in gastric cancer cells by regulating oxidative stress

**DOI:** 10.3389/fmolb.2025.1676811

**Published:** 2025-12-02

**Authors:** Zhenglei Ji, Lu Liu, Jingya Chen, Wanjing Zhu, Yunli Zhao, Huazhang Wu

**Affiliations:** 1 School of Public Health, Bengbu Medical University, Bengbu, Anhui, China; 2 School of Life Science, Anhui provincial key laboratory of tumor evolution and intelligent diagnosis and treatment, Bengbu, Anhui, China

**Keywords:** gastric cancer (GC), tumor necrosis factor-associated protein 1 (TRAP1), Oxidative stress, cisplatin (CDDP), drug resistance

## Abstract

**Introduction:**

Gastric cancer (GC) is a common malignancy of digestive system with high morbidity and mortality. Cisplatin (CDDP) is often applied in GC clinical treatment, particularly in the postoperative adjuvant chemotherapy, where it improves patient survival and reduces recurrence risk. However, the development of drug resistance following prolonged use poses an obstacle in its clinical use. This study investigated the role of tumor necrosis factor receptor-associated protein 1 (TRAP1) in modulating the sensitivity of GC cells to CDDP through oxidative stress pathway.

**Methods:**

Bioinformatic analysis was employed to assess TRAP1 expression in GC tissues compared to adjacent normal gastric tissues, and to evaluate its association with patient prognosis. Using lentivirus transfection and RNA interference, GC cell models with TRAP1 overexpression and silencing were established, then reactive oxygen species (ROS), mitochondrial membrane potential (MMP), DNA damage and cell death were measured following treatment with CDDP alone or in combination with antioxidant N-acetyl-L-cysteine (NAC).

**Results:**

Results indicated that TRAP1 was upregulated in GC tissues and elevated TRAP1 was related with poor prognosis. In GC cells exposed to CDDP, TRAP1 reduced ROS, stabilized MMP and mitigated DNA damage, leading to diminished cell death. TRAP1 overexpression potentiated the protective effects of NAC, while TRAP1 silencing counteracted the protective effects.

**Discussion:**

These findings indicated that TRAP1 attenuated CDDP sensitivity in GC cells by reducing cell death caused by CDDP-induced oxidative stress. TRAP1 represented a potential biomarker and a therapeutic target in GC treatment. This study provided a new strategy for improving the efficacy of CDDP-based chemotherapy through individualized treatment approaches.

## 1 Introduction

Gastric cancer (GC), a prevalent malignant tumor of digestive system, exhibits high morbidity and mortality rates with poor prognosis, posing a significant threat to human health (Sung et al., 2021). Global cancer statistics indicated that GC ranks fifth among the most common cancers worldwide in both incidence and mortality (Bray et al., 2024). According to registration data from National Cancer Center of China, GC was the third leading cause of malignant tumor deaths nationally in 2022 (Lyu et al., 2024). Because of the frequent absence of symptoms in early stages and limited popular screening, most patients present advanced stage at diagnosis, contributing to high mortality. Notably, GC related deaths in China accounted for about 43.9% of the global death (Yan et al., 2023).

Most GC patients experience poor prognosis due to late diagnosis. To improve therapeutic efficacy, surgery combined with chemotherapy or radiation therapy contributes the standard treatment for advanced GC (Yuan et al., 2024). Cisplatin (CDDP), a platinum-based chemotherapeutic agent widely used in malignancies including GC, particularly in postoperative adjuvant chemotherapy (Makovec, 2019), can improve patient survival and reduce recurrence risk (Smyth et al., 2020). CDDP-induced toxicity involves multiple mechanisms, such as DNA damage, mitochondrial dysfunction, and oxidative stress (Siddik, 2003). However, drug resistance following prolonged administration poses a major clinical limitation (Amable, 2016). Therefore, exploring strategies to overcome CDDP resistance of GC presents an urgent priority for preclinical studies and clinical trials (Fu et al., 2023).

Existing evidences have proved that oxidative stress is associated with tumor chemoresistance (Maiti, 2010; Park et al., 2013; Zhang et al., 2018). Tumor necrosis factor receptor-associated protein 1 (TRAP1), a mitochondrial-localized molecular chaperone belongs to heat shock proteins HSP90 family, serves as type I protein of tumor necrosis factor (TNF) receptor (Song et al., 1995; Felts et al., 2000). Studies have confirmed that TRAP1 is closely related to maintaining mitochondrial homeostasis and regulating cell proliferation and apoptosis, particularly under oxidative stress (Guzzo et al., 2014).

ROS-mediated oxidative stress constitutes a cornerstone of carcinogenesis (Phull et al., 2024). Additionally, it induces cellular damage and apoptosis through cascading damage to proteins, lipids, and nucleic acids, highlighting its dual role in both tumor progression and cell fate regulation (Cheung and Vousden, 2022; Semenza, 2017; Attique et al., 2025; Manogaran et al., 2019). Mechanistically, ROS generate mitochondrial apoptotic pathway through: altered BAX/Bcl-2 expression, MMP alteration, cytochrome c release, and caspase cascade activation (Morse et al., 2024; Kalai Selvi et al., 2017). The mitochondrial chaperone protein TRAP1 has been reported in modulating ROS-mediated apoptosis regulation in tumor cells (Cui et al., 2018; Bruno et al., 2024; Masuda et al., 2004), while oxidative stress and mitochondrial dysfunction have been established as key factors in platinum-based chemotherapy mechanisms (McQuade et al., 2018). However, the special role of TRAP1 in GC remains poorly characterized, thus this study aims to explore whether TRAP1 affects CDDP resistance through oxidative stress regulation in GC cells.

Our research demonstrated that TRAP1 was upregulated in GC tissues and its elevated level correlated with poor patient prognosis. TRAP1 over-expression and silence GC cell models were established to investigate the association of TRAP1 and GC, and ROS, MMP, DNA damage and cell death were detected under CDDP treatment. To verify if TRAP1 protected GC cells via the oxidative stress pathway, the antioxidant N-acetyl-L-cysteine (NAC) was used to clear excessive ROS in GC cells. Results showed that NAC could weaken the damaging effects of TRAP1 silencing, and TRAP1 reduced the sensitivity of GC cells to CDDP by inhibiting CDDP-induced ROS production. Consequently, TRAP1 may be used as a potential biomarker in GC treatment, and this study proposes a new strategy for the individualized treatment by improving the sensitization to CDDP-based chemotherapy.

## 2 Materials and methods

### 2.1 Bioinformatics analysis

Gene expression matrix was conducted using R (version 4.4.1) and Perl (version 5.34.0) based on stomach adenocarcinoma (STAD) data from the Cancer Genome Atlas (TCGA) database (https://portal.gdc.cancer.gov/). Comparative analysis of TRAP1 expression between GC tissues and adjacent normal tissues was conducted using online tool GEPIA2 (http://gepia2.cancer-pku.cn/). Survival prognosis of GC patients based on TRAP1 expression levels (stratified by median expression) was analyzed with online tool Kaplan-Meier Plotter (https://kmplot.com/analysis/).

### 2.2 Proteomics analysis

Protein was extracted from each sample by SDT lysis (4% SDS, 100 mM Tris-HCl, pH 7.6). After quantification via the BCA method, 15 μg of protein was mixed with 5× loading buffer and denatured by heating in a boiling water bath for 5 min. SDS-PAGE electrophoresis was then conducted on 4%–20% precast gradient gel at a constant voltage of 180 V for 45 min, and the gel was subsequently stained with Coomassie Brilliant Blue R-250.

After trypsin digestion using the filter-aided sample preparation (FASP) method, the peptides were desalted using a C18 cartridge, lyophilized, reconstituted in 0.1% formic acid solution. The peptide concentration was determined by measuring the absorbance at 280 nm (OD_280_). An appropriate amount of iRT standard peptides was then added to each sample. Chromatographic separation was achieved using a nano-flow Vanquish Neo system, followed by data-independent acquisition (DIA) on an Astral high-resolution mass spectrometer (Thermo Fisher Scientific, United States). The mass spectrometer was operated in positive ion mode with the following settings: precursor ion scan range of 380–980 m/z and an MS1 resolution of 240,000 at 200 m/z. DIA data were processed using DIA-NN software with the following parameters: enzyme (trypsin), max missed cleavage site (Sung et al., 2021), fixed modification Carbamidomethyl (C), variable modifications Oxidation (M) and Acetyl (Protein N-term), and filtering parameter (FDR <1%).

Gene Ontology (GO) annotation of the target protein set was conducted using Blast2GO (BLASTP 2.8.0+), and Kyoto Encyclopedia of Genes and Genomes (KEGG) pathway annotation was performed using KOBAS software (Version 3.0). Fisher’s exact test (*P* < 0.05) was used to assess the significance of differences and identify the enriched pathway categories. Visualization was achieved using the clusterProfiler package in R (version 4.4.1) to generate KEGG pathway enrichment bubble plots and GO functional annotation bar charts.

### 2.3 Cell culture

GC cell lines used in this study included AGS and HGC-27, both were acquired from the Chinese Academy of Medical Sciences & Peking Union Medical College (CAMS and PUMC, China). Cells were incubated at 37 °C in a 5% CO_2_ incubator, using DMEM medium (GIBCO, United States) with 10% fetal bovine serum (Wisent, Canada), and 1% streptomycin-penicillin (Invitrogen, United States).

### 2.4 Construction of TRAP1 over-expressed and silenced GC cells

The AGS cell line was selected for TRAP1 overexpression. Cells were transfected with lentiviral vector using Hitrans G A viral infection enhancer (GenePharma Co., Ltd., Shanghai, China) when the cell confluence reached approximately 40%–50% density. Following 48 h incubation, stable transfectants were selected with 3.5 μg/mL of puromycin (Beyotime, China).

HGC-27 cell line was chosen for TRAP1 knockdown. Lipo 6000 was used for siRNA transfection when cell density reached 40%–50% (Beyotime, China) for 4 h–6 h (Jia et al., 2022). The sequences of TRAP1 siRNA #1 and #2 were 5′-AAUUCUGUGUCCUCGGAGGAC-3′ and 5′-AAACAUGAGUUCCAGGCCGAG-3′ respectively, with the sequence of siRNA as control: 5′-UUCUCCGAACGUGUCACGUTT-3′ (GenePharma Co., Ltd., Shanghai, China). The efficiencies of the TRAP1 knockdown and over-expression were verified by Western blot analysis (Supplementary Figure S1).

### 2.5 CCK-8 assay

Cell viability was assessed by CCK-8 assay. Cells were treated with CDDP or NAC for 24 h, then CCK-8 assay (Beyotime, China) was added followed by incubation for 3 h (Zhai et al., 2020). The optical density (OD) was detected at 450 nm by multimode microplate reader (Thermo Fisher Scientific, United States).

### 2.6 Detection of ROS

ROS in AGS cells and HGC-27 cells were assessed with dihydroethidium (DHE) (Pulilai, China) and 2′,7′-dichlorodihydrofluorescein diacetate (DCFH-DA) (Beyotime, China) respectively. Cells in the logarithmic growth phase were seeded in 6-well plates and cultured for 24 h, then CDDP was added for treatment. After treatment with CDDP for 24 h, cells were collected and washed twice with PBS followed by incubation with DHE or DCFH-DA at 37 °C for 30 min without light (Guo et al., 2022). After staining, cells were washed with PBS and loaded onto CytoFLEX flow cytometer (Beckman Coulter, United States) for detection or observed under inverted fluorescence microscope (Olympus, Japan). Data obtained from flow cytometer were analyzed using Flow Jo software (V.10.8.1), while fluorescent images were captured and analyzed using ImageJ software (1.8.0).

### 2.7 Detection of MMP

MMP was detected with Mito-Tracker Red CMXRo for AGS cells and JC-1 solution (Beyotime, China) in HGC-27 cells. After treatment with CDDP for 24 h, cells were collected and washed twice with PBS, followed by incubation with staining solution for 30 min. Then cell samples were loaded onto CytoFLEX flow cytometer (Beckman Coulter, United States) or observed under inverted fluorescence microscope (Olympus, Japan) after washed with PBS. Data from flow cytometer were obtained and analyzed with Flow Jo software (V.10.8.1), and fluorescent images were captured and analyzed using ImageJ software (1.8.0).

### 2.8 DNA damage assay

DNA damage was marked by γ-H2AX immunofluorescence kit (Beyotime, China). Cells were collected and fixed in accordance with protocol. Subsequently, cells were cultured with anti-rabbit γ-H2AX antibody and the nuclear staining solution 4, 6-Diamidino-2-phenylindole dihydrochloride (DAPI) overnight. Photos were captured under fluorescence microscope and immunofluorescence analysis was conducted by ImageJ software (1.8.0).

### 2.9 Cell death detection

Cell death was detected using flow cytometry. Cells were harvested and washed with PBS after treated with CDDP for 24 h. For AGS cells, 195 μL of binding buffer, 5 μL of Annexin V-fluorescein isothiocyanate (FITC) and 10 μL of propidium iodide (PI) (Beyotime, China) were added, and cells were incubated at 25 °C for 30 min without light. For HGC-27 cells, 100 μL of diluted Annexin V Binding Buffer, 2.5 μL of Annexin V-PE and 2.5 μL of 7-AAD (100 μg/mL) (Procell, China) were added and gently mixed, followed by incubation at room temperature in the dark for 30 min. Death cells (apoptosis and necrotic cells) were quantified using CytoFLEX flow cytometer (Beckman Coulter, United States), and data analysis was performed with Flow Jo software (V 10.8.1).

### 2.10 Western blot analysis for protein expression

Total protein was extracted using RIPA lysis buffer (Beyotime, China) and quantified via bicinchoninic acid (BCA) (Beyotime, China). Samples were then denatured by boiling at 100 °C for 5 min, resolved on 12.5% sodium dodecyl sulfate-polyacrylamide gel electrophoresis (SDS-PAGE) (Epizyme Biotech, China), and electrophoretically transferred to polyvinylidene difluoride (PVDF) membranes (EMD Millipore, United States).

PVDF membranes were washed three times with TBST (Tris-buffered saline containing 0.1% Tween-20) and blocked with 5% skim milk for 2 h at room temperature. After incubation with primary antibodies at 4 °C overnight, membranes were washed and probed with horseradish peroxidase (HRP)-conjugated secondary antibodies: Goat anti-Mouse IgG or Goat anti-Rabbit IgG (1:4000) (Proteintech, China) for 1.5 h at room temperature. Protein bands were visualized with enhanced chemiluminescence (ECL) HRP substrate kit (EMD Millipore) and imaged with the Image Lab software 6.1 (Bio-Rad Laboratories, United States).

### 2.11 Statistical analyses

Data are expressed as mean ± SEM of 3 independent experiments. Differences among groups were performed with ANOVA (three or more groups) or Student’s t-test (two groups) using SPSS 25.0 software (IBM Corporation, United States). Kaplan-Meier analysis was evaluated by log-rank test. Graphs were generated by GraphPad Prism 8.0. *P* < 0.05 indicates significance.

## 3 Results

### 3.1 TRAP1 was upregulated in GC tissues and forecasted adverse prognosis

**FIGURE 1:**
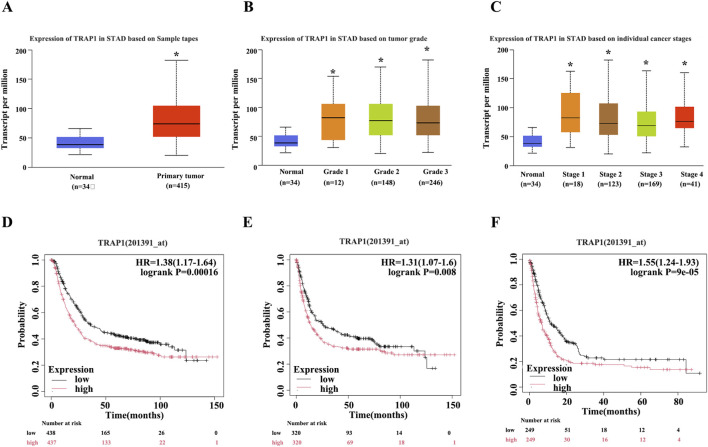
TRAP1 was upregulated in GC tissues and related with adverse prognosis for GC patients. **(A)** TRAP1 was significantly upregulated in GC tissues. **(B,C)** TRAP1 expression was independent of clinicopathological characteristics including tumor stage **(B)** and differentiation grade **(C)** in GC patients. **(D–F)** Kaplan-Meier survival curves of OS **(D)**, FP **(E)**, and PPS **(F)** in GC patients. OS: overall survival, FP: survival to first progression, PPS: post progression overall survival; *: *P* < 0.05; **: *P* < 0.01; ***: *P* < 0.001 compared with normal gastric tissue.

To explore the underlying function of TRAP1 in GC, the relative expressions of TRAP1 in GC and adjacent normal tissues were analyzed with TCGA database. Results from TCGA database analysis indicated that TRAP1 was remarkably increased in GC tissues compared with adjacent normal tissues (*P* < 0.05, Figure 1A; Supplementary Figure S2). Furthermore, the upregulation was independent of clinicopathological characteristics, such as tumor stage and differentiation grade in GC patients (Figures 1B,C).

Survival analysis with K-M Plotter demonstrated that GC patients with elevated TRAP1 exhibited poorer prognosis, including reduced overall survival (OS, HR = 1.38, *P* < 0.01), first progression survival (FP, HR = 1.31, *P* < 0.01) and post progression overall survival (PPS, HR = 1.55, *P* < 0.01) (Figures 1D–F). Collectively, upregulated TRAP1 in GC tissue constitutes an independent prognostic factor, indicating that it may serve as a promising therapeutic target and forecast biomarker.

### 3.2 TRAP1 regulated oxidative stress process in GC cells

**FIGURE 2:**
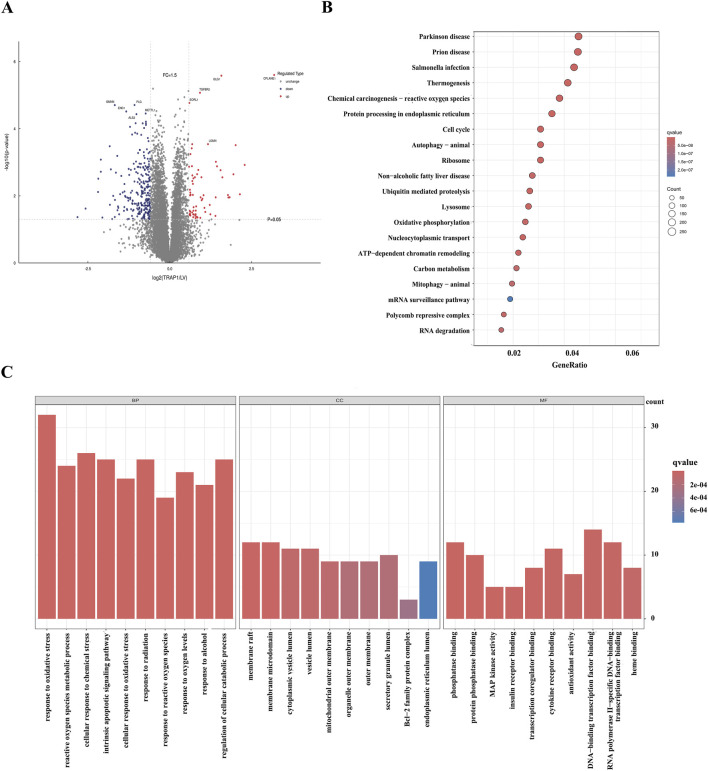
Proteomic analysis of the overexpression of TRAP1 in GC cells. **(A)** The volcano plot shows DEPs in TRAP1 over-expressed GC cells. **(B)** KEGG analysis of DEPs. **(C)** GO functional analysis of the DEPs. DEPs: Differentially expressed proteins.

To explore the role of TRAP1 in CDDP resistance, this study performed proteomics analysis in TRAP1 overexpressed AGS cells. 371 DEPs were identified (|log2 FC|>1.5 and *P* < 0.01), with 99 upregulated and 272 downregulated (Figure 2A). KEGG pathway analysis indicated that these genes participated in the cellular response to oxidative stress, ROS metabolism processes, and mitochondrial membrane function (Figure 2B). GO enrichment revealed that these significant DEPs were enriched in multiple pathways, including chemical carcinogenesis-reactive oxygen species, oxidative phosphorylation, and DNA damage repair signaling pathways (Figure 2C). Integrated with existing literature, these proteomics findings suggest that TRAP1 mitigates cell death through oxidative stress regulation in GC cells.

### 3.3 TRAP1 reduced cell death by regulating oxidative stress in GC cells

#### 3.3.1 TRAP1 reduced ROS levels in GC cells

**FIGURE 3:**
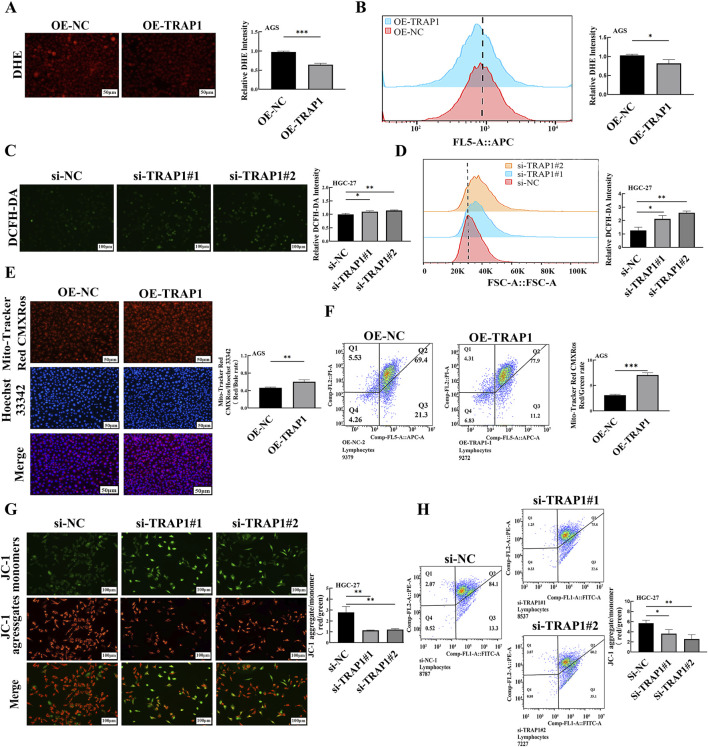
TRAP1 reduced ROS levels and maintained MMP in GC cells. **(A,B)** ROS levels in TRAP1 over-expressed AGS cells stained by DHE and detected by FCM. **(C,D)** Accumulation of ROS in TRAP1 silenced HGC-27 cells stained by DCFH-DA and detected by FCM **(E,F)** MMP in TRAP1 over-expressed AGS cells stained with Mito-Tracker Red CMXRos and detected by FCM. **(G,H)** MMP in TRAP1 silenced HGC-27 cells stained with JC-1 stain and detected by FCM. NC: negative control; OE: over expression; Si:silence. *: *P* < 0.05; **: *P* < 0.01; ***: *P* < 0.001 vs. control.

Oxidative stress induced by CDDP and other chemotherapy drugs is the primary mechanism triggering cancer cell apoptosis. However, the clearance of intracellular ROS enables cells to evade oxidative stress induced apoptosis, ultimately resulting in chemoresistance. To explore the function of TRAP1 on ROS regulation, we established TRAP1 over-expressed and silenced GC cells with AGS and HGC-27 cells, and transfection efficiencies were shown in Supplementary Figure S1. In TRAP1 over-expressed AGS cells, DHE staining demonstrated attenuated fluorescence intensity of ROS (Figure 3A), and similar results were also obtained through flow cytometry (FCM) analysis (Figure 3B). On the contrary, HGC-27 cells with silenced TRAP1 exhibited augmented ROS fluorescence intensity (Figure 3C) and elevated accumulation of ROS by FCM analysis (Figure 3D). These findings suggest that TRAP1 over-expression significantly suppresses ROS production whereas TRAP1 silencing promotes ROS accumulation in GC cells.

#### 3.3.2 TRAP1 maintained MMP in GC cells

Mitochondrion is a vital target of oxidative stress, where intracellular ROS accumulation disrupts MMP and triggers apoptosis. We assessed the impacts of TRAP1 on mitochondrial integrity in GC cells using Mito-Tracker Red CMXRos and JC-1 probes. Compared with control, increased red fluorescence intensities were found in TRAP1 over-expressed AGS cells under microscope (Figure 3E), which were consistent with FCM results (Figure 3F). Conversely, enhanced green fluorescence intensity was found in TRAP1 silenced HGC-27 cells (Figure 3G), indicating reduced MMP (Figure 3H). Hence, TRAP1 maintained the mitochondrial function and MMP by alleviating ROS accumulation, which in turn promoted GC progression.

#### 3.3.3 TRAP1 mitigated DNA damage in GC cells

**FIGURE 4:**
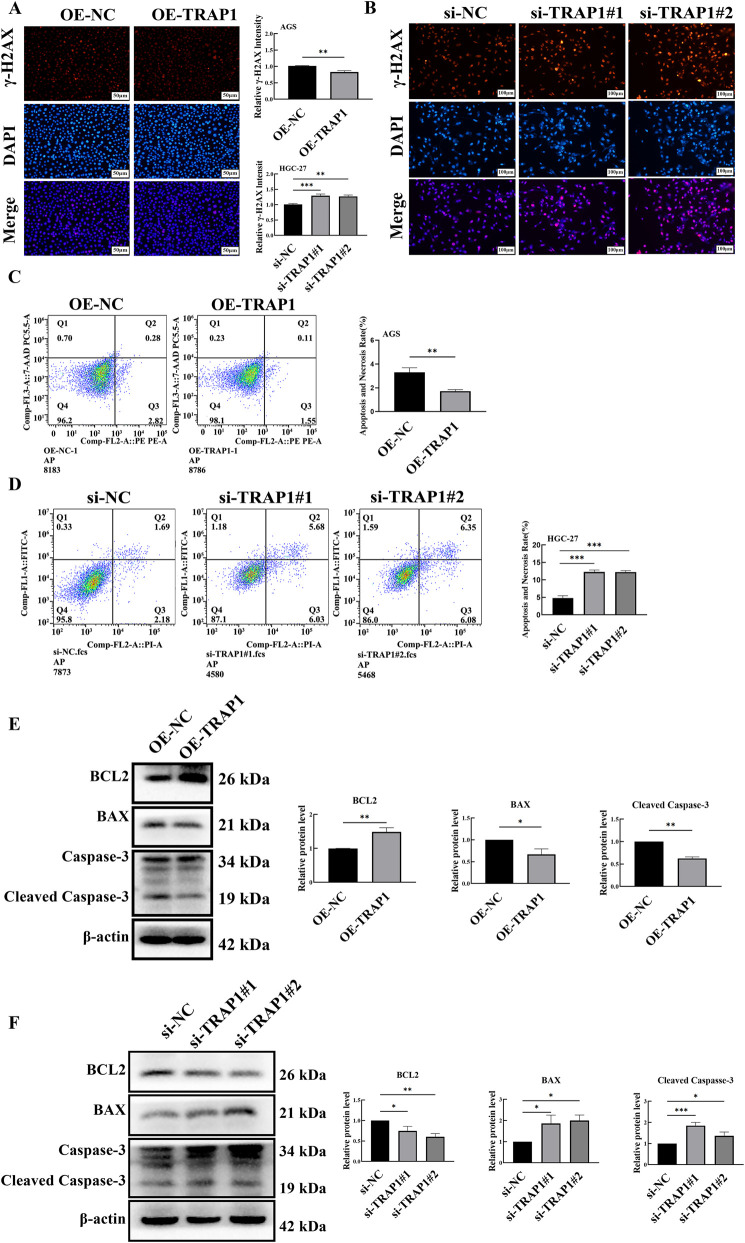
TRAP1 suppressed DNA damage and cell death in GC cells. **(A)** TRAP1 over-expression reduced γ-H2AX in GC cells. **(B)** TRAP1 silencing increased γ-H2AX in HGC-27 cells. **(C,D)** Cell death detected by FCM in TRAP1 over-expressed AGS cells and TRAP1 silenced HGC-27 cells. **(E,F)** Western blot analysis of the expression levels of apoptosis related proteins in TRAP1-overexpressed AGS cells and TRAP1-silenced HGC-27 cells. NC: negative control; OE: over expression; Si:silence. *: *P* < 0.05; **: *P* < 0.01; ***: *P* < 0.001 vs. control.

Since DNA damage represents both a consequence of oxidative stress and a primary inducer of apoptosis, we assessed the canonical DNA damage biomarker γ-H2AX in GC cells. Results from immunofluorescence microscope indicated that TRAP1 over-expression reduced γ-H2AX foci formation in GC cells (Figure 4A), whereas TRAP1 silencing increased γ-H2AX phosphorylation levels (Figure 4B). These findings indicated that TRAP1 suppressed DNA damage.

#### 3.3.4 TRAP1 inhibited cell death of GC cells

Given that death is the dominating endpoint of oxidative stress, we evaluated the influence of TRAP1 on death by Annexin V staining (PE/7-AAD for AGS cells and FITC/PI for HGC-27 cells). FCM quantification revealed a reduced cell death rate in TRAP1 over-expressed AGS cells (Figure 4C), but TRAP1 silenced HGC-27 cells exhibited increased cell death (Figure 4D). These results demonstrated that TRAP1 inhibited cell death in GC cells.

#### 3.3.5 TRAP1 modulated cell death related proteins

To reveal the mechanism underlying TRAP1-mediated CDDP resistance in GC cells, we detected the expression levels of apoptosis-related proteins. Western blot analysis revealed that TRAP1 overexpression significantly upregulated the anti-apoptotic protein Bcl-2, while downregulating the pro-apoptotic protein BAX and Cleaved Caspase-3 (Figure 4E). Conversely, in TRAP1 silenced HGC-27 cells, Bcl-2 exhibited significant downregulation, while BAX and Cleaved Caspase-3 showed marked upregulation (Figure 4F). These results suggested that TRAP1 regulated apoptosis signaling through coordinated modulation of Bcl-2 and caspase activation.

### 3.4 TRAP1 combined with NAC in inhibiting the accumulation of ROS and cell death in GC cells

#### 3.4.1 TRAP1 combined with NAC in inhibiting ROS in GC cells

**FIGURE 5:**
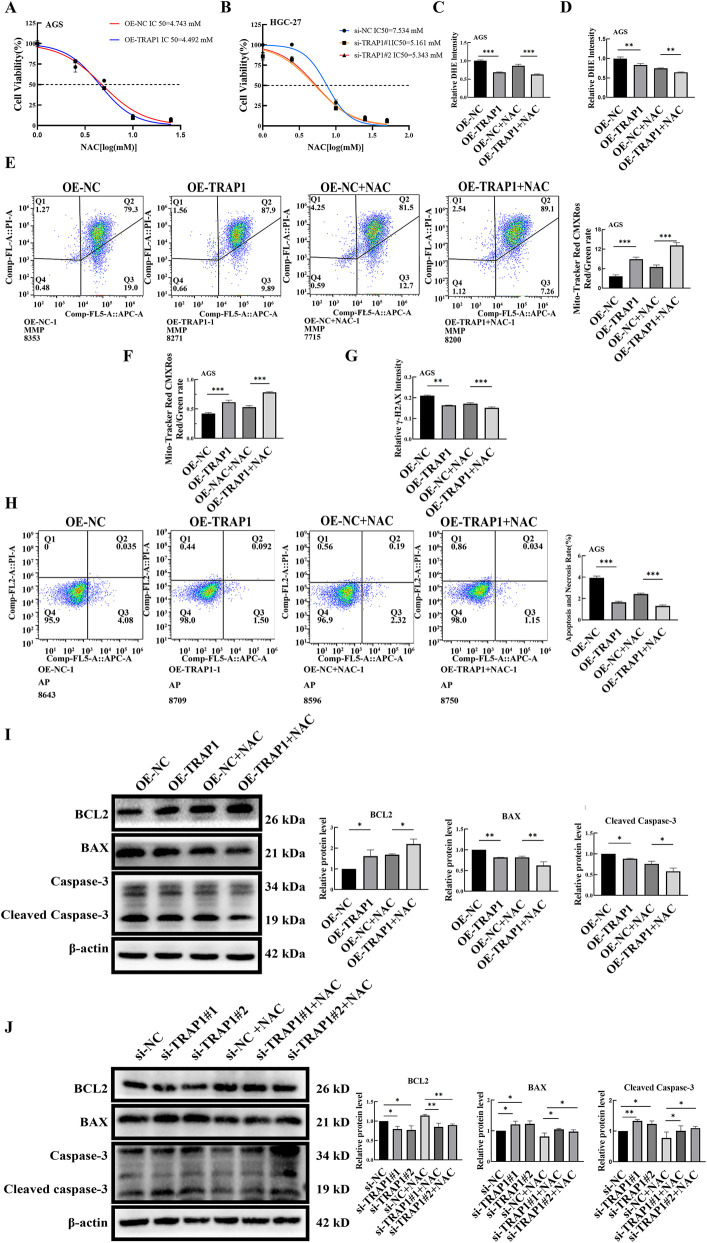
The combination of TRAP1 and NAC inhibits the accumulation of ROS and cell death in GC cells. **(A,B)** Cell viability under NAC treatment in TRAP1 over-expressed AGS cells and TRAP1 silenced HGC-27 cells. **(C,D)** The combined use of TRAP1 and NAC further attenuated DHE fluorescence staining and detected by FCM in AGS cells. **(E,F)** In AGS cells co-treated with TRAP1 and NAC, MMP was labeled using Mito-Tracker Red CMXRos and analyzed by FCM. **(G)** Combination of NAC and TRAP1 reduced the level of DNA damage marker γ-H2AX in GC cells. **(H)** Combination of TRAP1 and NAC inhibited cell death in GC cells. **(I,J)** Protein expressions following NAC treatment in AGS and HGC-27 cells. NC: negative control; OE: over expression; Si:silence. *: *P* < 0.05; **: *P* < 0.01; ***: *P* < 0.001 vs. control.

To verify whether TRAP1 modulates cellular characters through oxidative stress, GC cells were treated with ROS scavenger NAC, followed by assessment of ROS accumulation and apoptosis. GC cells underwent NAC treatment at varying concentrations, and IC_50_ was determined by CCK-8 assay. In GC cells, TRAP1 over-expression increased the IC_50_ of NAC, whereas TRAP1 silencing decreased the IC_50_ (Figures 5A,B). Based on the calculated IC_50_, subsequent experiments applied 2.5 μM and 3.5 μM NAC for AGS cells and HGC-27 cells, respectively.

Fluorescence microscopy observation showed that NAC treatment further reduced the relative fluorescence intensity of ROS in TRAP1 over-expressed AGS cells, and NAC significantly antagonized the increase in ROS induced by TRAP1 silencing in HGC-27 cells (Figure 5C; Supplementary Figure S3A,B). Consistent alterations in ROS were also found by FCM (Figure 5D; Supplementary Figure S3C,D).

#### 3.4.2 TRAP1 and NAC maintained the MMP level in GC cells

We next examined whether TRAP1 collaborated with NAC to maintain MMP in GC cells. Results of FCM showed a cooperation between TRAP1 over-expression and NAC in maintaining higher MMP in AGS cells (Figure 5E). Conversely, NAC restored the MMP reduction induced by TRAP1 silencing (Supplementary Figure S3E). Under fluorescence microscopy, AGS cells with over-expressed TRAP1 showed elevated red fluorescence after being treated with NAC, when compared to either TRAP1 over-expressed or NAC treated control AGS cells (Figure 5F; Supplementary Figure S3H). On the contrary, NAC treatment rescued the decrease in green fluorescence caused by TRAP1 silencing in HGC-27 cells (Supplementary Figure S3F,G). Collectively, TRAP1 cooperated with NAC in inhibiting MMP decrease, and NAC counteracted the MMP attenuation in TRAP1 silenced GC cells.

#### 3.4.3 Combined action of TRAP1 and NAC in reducing DNA damage

Based on above findings, we can infer that TRAP1 could mitigate DNA damage in GC cells. Consequently, we assessed the DNA damage marker γ-H2AX. In AGS cells, TRAP1 over-expression combined with NAC significantly reduced γ-H2AX expression compared to either TRAP1 over-expression or NAC treatment alone (Figure 5G; Supplementary Figure S4A). In HGC-27 cells, TRAP1 silencing increased γ-H2AX and NAC reversed the increase (Supplementary Figure S4B,C). These observations suggest that TRAP1 cooperated with NAC in inhibiting DNA damage, which may regulate cell death through oxidative stress and DNA damage in GC cells.

#### 3.4.4 TRAP1 combined with NAC in reducing cell death and regulating apoptosis-related proteins in GC cells

To further explore whether TRAP1-mediated DNA protection inhibited cell death in GC cells, cell death was assessed by FCM. Results showed that both NAC treatment and TRAP1 overexpression reduced cell death compared to TRAP1 overexpression or NAC treatment alone (Figure 5H). In HGC-27 cells, NAC treatment restored TRAP1 silencing-induced cell death (Supplementary Figure S4D). These results indicated that both TRAP1 and NAC could alleviate DNA damage by alleviating oxidative stress and inhibiting cell death in GC cells.

Following NAC treatment in TRAP1 overexpressed GC cells, the expression level of Bcl-2 increased, whereas BAX and Cleaved Caspase-3 were significantly decreased (Figure 5I). This result indicated that the combined action of TRAP1 and NAC enhanced cellular anti-apoptotic ability. Similarly, in TRAP1-silenced GC cells, NAC treatment led to a modest upregulation of Bcl-2, accompanied by a slight downregulation of BAX and Cleaved Caspase-3 (Figure 5J). These results indicated that TRAP1, analogous to NAC, regulates the expression of BAX, Bcl-2, and Cleaved Caspase-3 through oxidative stress, thereby modulating cell death.

### 3.5 TRAP1 reduced CDDP-induced ROS and cell death in GC cells

#### 3.5.1 TRAP1 reduced CDDP-induced ROS accumulation

**FIGURE 6:**
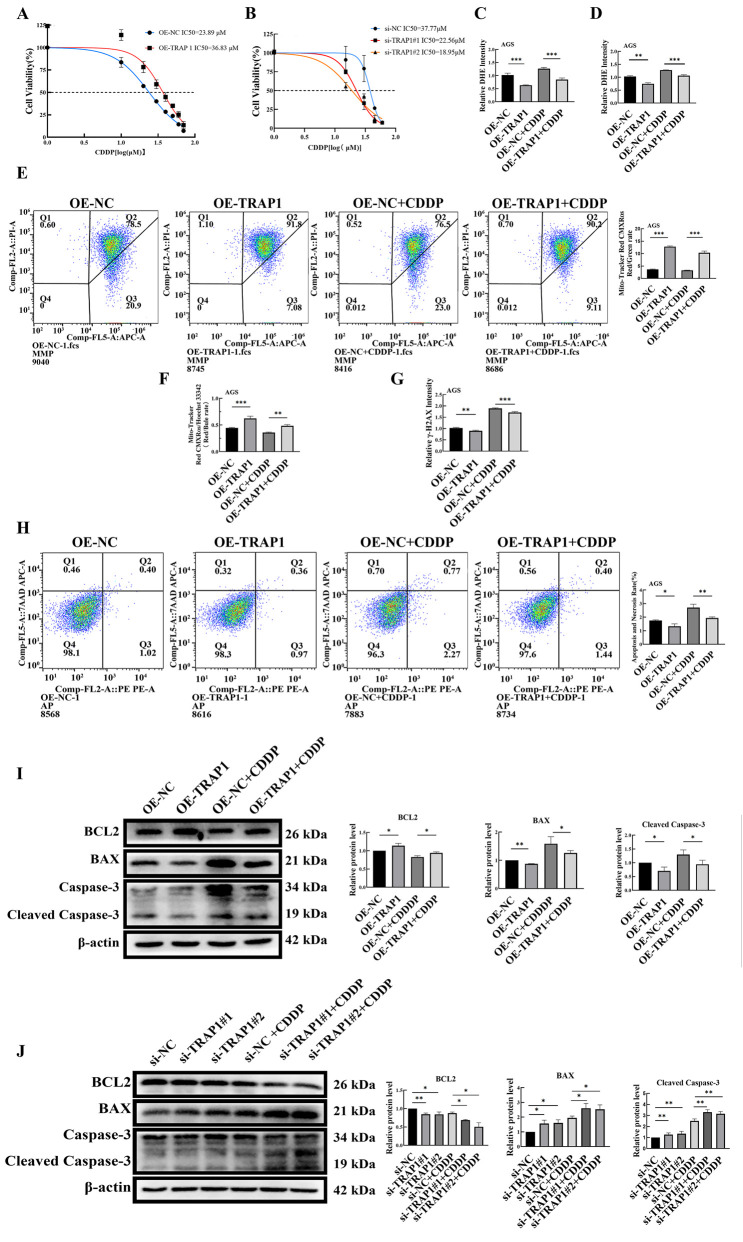
TRAP1 reduces the level of ROS and inhibits cell death in CDDP-induced GC cells. **(A,B)** TRAP1 overexpression reduces the sensitivity of AGS cells to CDDP, TRAP1 silencing enhances the sensitivity of HGC-27 cells to CDDP. **(C,D)** Overexpression of TRAP1 attenuates DHE fluorescence staining and detected by FCM in CDDP-treated AGS cells. **(E,F)** MMP was detected by FCM and fluorescent probe in CDDP-treated AGS cells with TRAP1 overexpression. **(G)** TRAP1 overexpression reduces the level of DNA damage marker γ-H2AX induced by CDDP in AGS cells. **(H)** Over-expression TRAP1 inhibited CDDP caused cell death in GC cells. **(I,J)** Apoptosis-related protein expression in AGS and HGC-27 cells after CDDP treatment. NC: negative control; OE: over expression; Si: silence. *: *P* < 0.05; **: *P* < 0.01; ***: *P* < 0.001 vs. control.

During CDDP chemotherapy, cancer cells adapt by adjusting intracellular ROS levels, ultimately leading to CDDP resistance. To investigate whether TRAP1 influenced the sensitivity of GC cells to CDDP, CCK-8 assay was performed to evaluate IC_50_ of CDDP in GC cells. Results demonstrated that TRAP1 over-expression significantly reduced CDDP sensitivity yielding a higher IC_50_ value, whereas TRAP1 silencing enhanced CDDP sensitivity in HGC-27 cells with a lower IC_50_ (Figures 6A,B).

Based on calculated IC_50_ values, 20 μM and 15 μM CDDP were selected for AGS and HGC-27 cells, respectively. Under CDDP treatment, TRAP1 over-expressed AGS cells exhibited lower ROS levels compared to control AGS cells, though their ROS levels remained higher than those without CDDP treatment (Figure 6C; Supplementary Figure S5A). Conversely, CDDP treatment elevated ROS levels in TRAP1 silenced HGC-27 cells compared with non-treated or wild type HGC-27 controls (Supplementary Figure S5B). Results from FCM confirmed same trends (Figure 6D; Supplementary Figure S5C,D). These findings suggested that TRAP1 antagonized CDDP-induced oxidative stress to confer chemoresistance in GC cells.

#### 3.5.2 TRAP1 antagonized CDDP-induced MMP reduction in GC cells

MMP was subsequently assessed in GC cells. In CDDP-treated TRAP1 over-expressing AGS cells, MMP was lower than untreated TRAP1 over-expressed control, but higher than CDDP treated wild-type AGS cells (Figure 6F; Supplementary Figure S5H). On the contrary, CDDP treated TRAP1-silencing HGC-27 cells showed a dramatic decrease in MMP than untreated wild-type control or TRAP1 silenced control (Supplementary Figure S5F,G). Consequences of FCM were in line with the findings of fluorescence probe analysis. Based on the findings above, TRAP1 sustained MMP integrity, whereas TRAP1 silencing exacerbated CDDP-induced MMP loss in GC cells (Supplementary Figure S5E) (Figure 6E).

#### 3.5.3 TRAP1 antagonized CDDP-induced DNA damage

Since the elevated intracellular ROS induce DNA damage and subsequent apoptosis, these endpoints were measured in CDDP treated GC cells. CDDP treatment increased DNA damage in AGS cells, while TRAP1 over-expression partly attenuated this damage (Figure 6G; Supplementary Figure S6A). In HGC-27 cells, both TRAP1 silencing and CDDP treatment aggravated DNA damage, with maximal damage observed in CDDP treated TRAP1 silenced cells (Supplementary Figure S6B,C). Taken together, TRAP1 mitigated CDDP-induced DNA damage in GC cells.

#### 3.5.4 TRAP1 reduced CDDP-induced cell death by regulating the expressions of apoptosis-related proteins

Cell death levels in TRAP1 overexpression and silencing GC cells treated with CDDP were detected using FCM. In AGS cells, TRAP1 overexpression attenuated CDDP-induced cell death (Figure 6H). In HGC-27 cells, TRAP1 silencing combined with CDDP treatment enhanced cell death compared to TRAP1 silencing or CDDP treatment alone (Supplementary Figure S6D). These results suggested that TRAP1 attenuated CDDP-induced DNA damage in GC cells and consequently suppressed cell death.

Further analysis revealed that CDDP treatment in TRAP1 overexpressed cells slightly downregulated Bcl-2 expression, with mildly increased BAX and Cleaved Caspase-3 (Figure 6I). In TRAP1 silenced cells, CDDP treatment promoted the decrease of Bcl-2, while significantly upregulated BAX and Cleaved Caspase-3 (Figure 6J). These results fully demonstrate that CDDP collaborated with TRAP1 in inducing cell death in GC cells by regulating BAX, Bcl-2, and Cleaved Caspase-3.

## 4 Discussion and conclusion

According to the Global Burden of Disease Study of 2022 data, China reported the highest incidence of new cancer cases and cancer-related deaths, posing a serious threat to public health (Bray et al., 2024). GC is a highly prevalent malignancy with elevated mortality rates, primarily attributable to late-stage diagnosis resulting from non-specific early-stage symptoms (Guan et al., 2023; Ajani et al., 2022). CDDP is widely used in chemotherapy for multiple malignancies including GC (Zangouei et al., 2021). However, chemotherapy resistance develops during clinical treatment. This development represents a major challenge in GC management (Al-Bahlani et al., 2020). Therefore, identifying therapeutic targets for overcoming CDDP resistance is an urgent clinical challenge in GC chemotherapy.

TRAP1, a member of the Hsp90 family and a TNF receptor-related protein (Song et al., 1995), regulates multiple cellular physiological processes including proliferation, differentiation and survival. Studies have demonstrated its critical involvement in oncogenesis (Masgras et al., 2021; Im, 2016). In this study, we analyzed the expression of TRAP1 in GC tissues using public databases. Consistent with Han’s findings (Han et al., 2016), the expression of TRAP1 in GC tissues was significantly higher than that in adjacent normal tissues (Figure 1A). Furthermore, high TRAP1 expression was correlated with poor prognosis (Figure 1B). Therefore, TRAP1 functions as a protective factor in GC progression.

TRAP1 is highly expressed in multiple malignancies including glioblastoma, colon cancer, breast cancer, prostate cancer and lung cancer, which is frequently associated with chemoresistance (Costantino et al., 2009; Sciacovelli et al., 2013; Lisanti et al., 2014; Zhang et al., 2015; Matassa et al., 2018). In esophageal squamous cell carcinoma, elevated TRAP1 expression antagonized CDDP-induced apoptosis, while its downregulation enhanced the CDDP sensitivity and promoted apoptosis (Ou et al., 2014). Moreover, researchers have demonstrated TRAP1’s involvement in lung cancer and colorectal cancer via mitochondria and ROS (Ou et al., 2014). Additionally, other GC-related studies have mostly focused on the relationship between other members of the heat shock protein family (e.g., HSP90AA1, HSP110) and platinum-based drug resistance, with few studies investigating the role of TRAP1 in CDDP resistance of GC cells (Li et al., 2025; Kimura et al., 2016).

Previous research has demonstrated that TRAP1 protects against oxidant-induced DNA damage and apoptosis (L et al., 2010; Hua et al., 2007), and functions as a ROS antagonist to suppress ROS production (L et al., 2010; Hua et al., 2007). In this study, our results corroborate these mechanisms: TRAP1 overexpression in GC cells reduced ROS levels, maintained mitochondrial function, attenuated DNA damage and suppressed apoptosis. Conversely, TRAP1 silencing induced ROS accumulation, diminished MMP, increased DNA damage and promoted apoptosis. Western blot analysis revealed that TRAP1 overexpression upregulated anti-apoptotic protein Bcl-2 while downregulated pro-apoptotic protein BAX and Cleaved Caspase-3. Concurrently, TRAP1 silencing produced reciprocal effects. These results indicated that TRAP1-mediated apoptosis inhibition contributed to chemoresistance in GC cells.

ROS, produced by cellular metabolism and environmental stimuli, regulate cellular homeostasis through modulation of intracellular oxygen environment (Jelic et al., 2021). In cancer cells, ROS play a core role in governing apoptosis, thereby modulating proliferation, survival and chemoresistance (Cui et al., 2018). CDDP induces apoptosis by elevating ROS. Research has proven that eliminating ROS could change the REDOX state in cancer cells, consequently altering their sensitivity to CDDP (Sobol et al., 2013). Thus, oxidative stress plays an important role in regulating cellular CDDP sensitivity.

To confirm whether TRAP1 suppresses apoptosis in GC cells by reducing ROS levels, we treated GC cells with the ROS scavenger NAC. Results showed that NAC reduced ROS generation, attenuated DNA damage and decreased apoptosis caused by TRAP1 silencing. In TRAP1 overexpressed GC cells, NAC treatment significantly upregulated Bcl-2 while downregulated BAX and Cleaved Caspase-3. Conversely, NAC rescued TRAP1 silencing-induced reduction in Bcl-2 expression, and suppressed the upregulation of BAX and Cleaved Caspase-3 (Figure 5). These findings indicated that both NAC and TRAP1 exerted protective effects by eliminating ROS and mitigating intracellular oxidative stress.

Studies have demonstrated that CDDP induces apoptosis by elevating free radical levels, particularly ROS (Mirzaei et al., 2021). Furthermore, oxidative damage has been confirmed as the main mechanism underlying CDDP cytotoxicity (Wang et al., 2022). As a key mitochondrial chaperone, TRAP1 exerts anti-apoptotic function through two interconnected pathways: oxidative stress and apoptosis (Montesano Gesualdi et al., 2007). Previous reports have shown that TRAP1 can directly interact with cytochrome c oxidase (Complex IV) and modulate its activity, thereby stabilizing electron transfer at the mitochondrial respiratory chain and reducing superoxide production (Yoshida et al., 2013). In line with reports, our results provide indirect support for the role of TRAP1 in reinforcing mitochondrial antioxidant defense system. Specifically, TRAP1 overexpression significantly attenuated CDDP-induced ROS accumulation, an effect potentially contributable to the upregulation of key antioxidant enzymes such as superoxide dismutase 2 (SOD2) and catalase (CAT) (Sciacovelli et al., 2013). SOD2 catalyzes the conversion of superoxide (O_2_•^-^) into hydrogen peroxide (H_2_O_2_), which is subsequently decomposed to water and oxygen by CAT. TRAP1 is likely to maintain the efficiency of this enzymatic antioxidant system, thereby collectively sustaining cellular redox homeostasis and counteracting CDDP-induced cytotoxicity (Ramos Rego et al., 2021; Faienza et al., 2020). Consistent with these observations, our findings demonstrated that TRAP1 confers CDDP resistance in GC cells through its antioxidative properties. Specifically, TRAP1 overexpression increased CDDP tolerance with significantly increased IC_50_, indicating enhanced drug tolerance, whereas TRAP1 silencing augmented CDDP effects by reducing MMP and aggravating DNA damage, thereby promoting cell apoptosis.

In the mitochondrial apoptotic pathway, the TRAP1-mediated reduction in oxidative stress further inhibited the activation of pro-apoptotic molecules such as BAX and Cleaved Caspase-3, and mitigated the suppression of the anti-apoptotic protein Bcl-2, thereby blocking the apoptotic cascade triggered by CDDP (Zhang et al., 2021; Xie et al., 2020). The results of this study provided supporting evidence. In CDDP-treated TRAP1-overexpressing GC cells, Bcl-2 was downregulated compared with the untreated overexpression control. Although BAX and Cleaved Caspase-3 showed a slight increase, their levels remained significantly lower than in the CDDP-treated wild-type control. This attenuation suggested that TRAP1 overexpression counteracted CDDP-induced pro-apoptotic signaling. Furthermore, TRAP1 silencing combined with CDDP treatment in reducing the expression of Bcl-2, while upregulating BAX and Cleaved Caspase-3 (Figure 6J). Therefore, TRAP1 protects GC cells from CDDP-induced apoptosis by alleviating oxidative damage, thereby promoting chemoresistance. In conclusion, our findings demonstrate that TRAP1 inhibits CDDP-induced apoptosis in GC cells by alleviating oxidative damage and protecting mitochondrial function, thereby conferring chemoresistance to GC cells.

One promising strategy to reverse chemoresistance involves the use of inhibitors targeting core drug-resistant factors. However, traditional broad-spectrum Hsp90 inhibitors lack isoform selectivity, resulting in concurrent inhibition of Hsp90 family proteins in normal cells. This off-target effect contributes to severe toxicity and has limited their clinical application (Reynolds et al., 2025). Our study confirms the involvement of TRAP1 in CDDP resistance in GC. As a member of the Hsp90 family, TRAP1 mediates drug resistance through the oxidative stress pathway (Yoshida et al., 2013). This insight highlights the potential of developing TRAP1-specific inhibitors, which may reverse CDDP resistance while minimizing systemic toxicity (Serapian et al., 2021).

Despite these advances, the precise mechanism by which TRAP1 interacts with downstream proteins remain unclear. Future studies should focus on identifying TRAP1-binding proteins using co-immunoprecipitation combined with mass spectrometry (Co-IP-MS), as well as assessing the influence of TRAP1 on the activity of key antioxidant enzymes. These investigations would strengthen the functional evidence supporting TRAP1 as a therapeutic target for overcoming drug resistance.

In summary, TRAP1 was highly expressed in GC tissues and correlated with poor prognosis. TRAP1 reduced the oxidative stress levels, preserved MMP, mitigated DNA damage and consequently inhibited cell death in GC cells. Crucially, TRAP1 contributed to CDDP resistance in GC cells by alleviating oxidative damage and regulating the expression of apoptosis-related proteins (Bcl-2, BAX, and Cleaved Caspase-3), which collectively inhibit apoptosis and promote chemoresistance.

## Data Availability

The datasets used and analyzed during the study are available from the corresponding author on reasonable request.
