# Supplementary material for: TRAP1 induced cisplatin resistance in gastric cancer cells by regulating oxidative stress
Source: Front Mol Biosci. 2025 Dec 2;12:1676811. doi: 10.3389/fmolb.2025.1676811 (PMC12705405; doi:10.3389/fmolb.2025.1676811)
Supplement: Supplementary file 1 [file DataSheet1.docx]

Supplementary Material

## Supplementary Figures


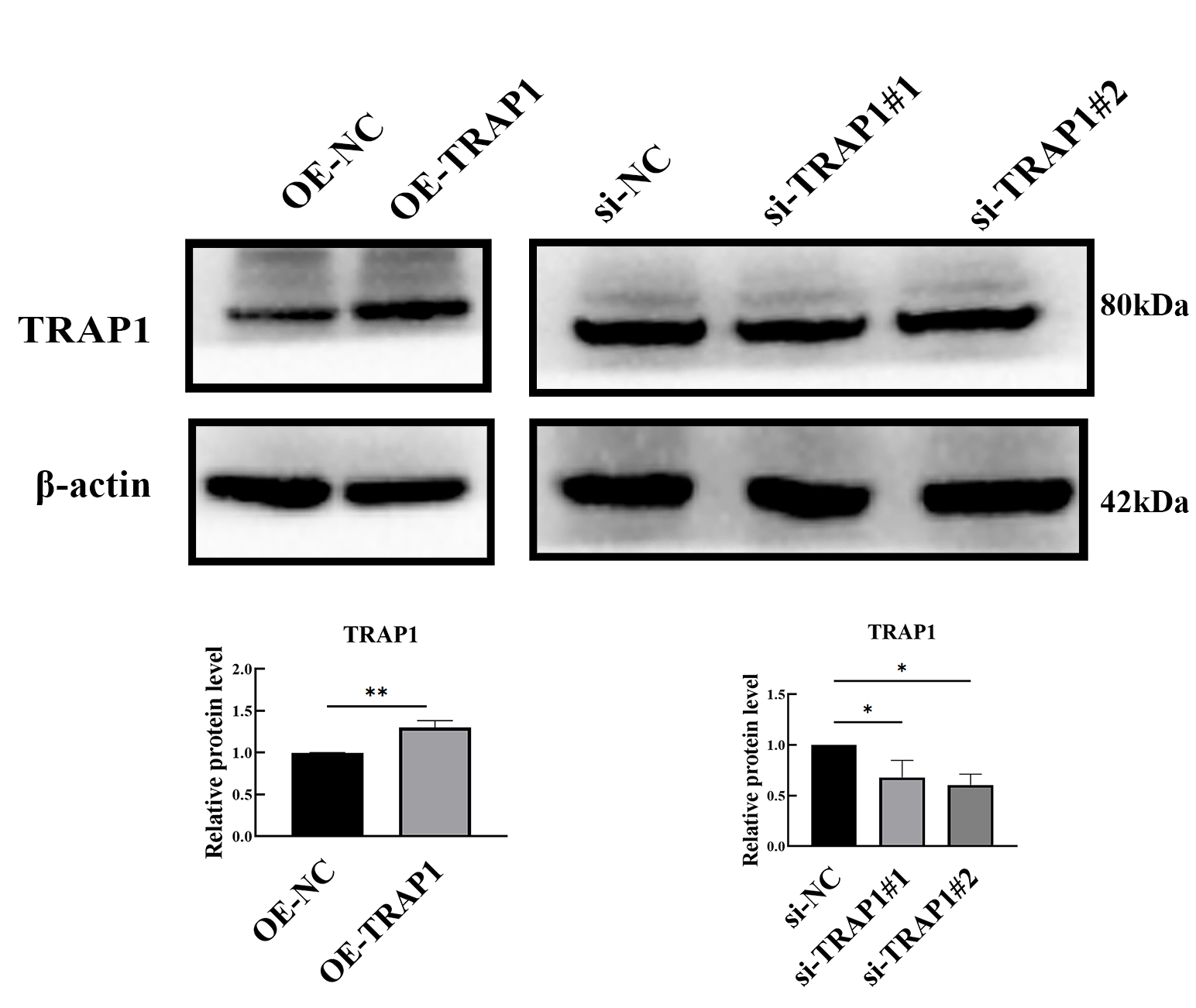


**Figure S1 TRAP1 overexpression and silencing efficiency was analyzed by western blotting in GC cells.** NC: negative control; OE: over expression; Si: silence.


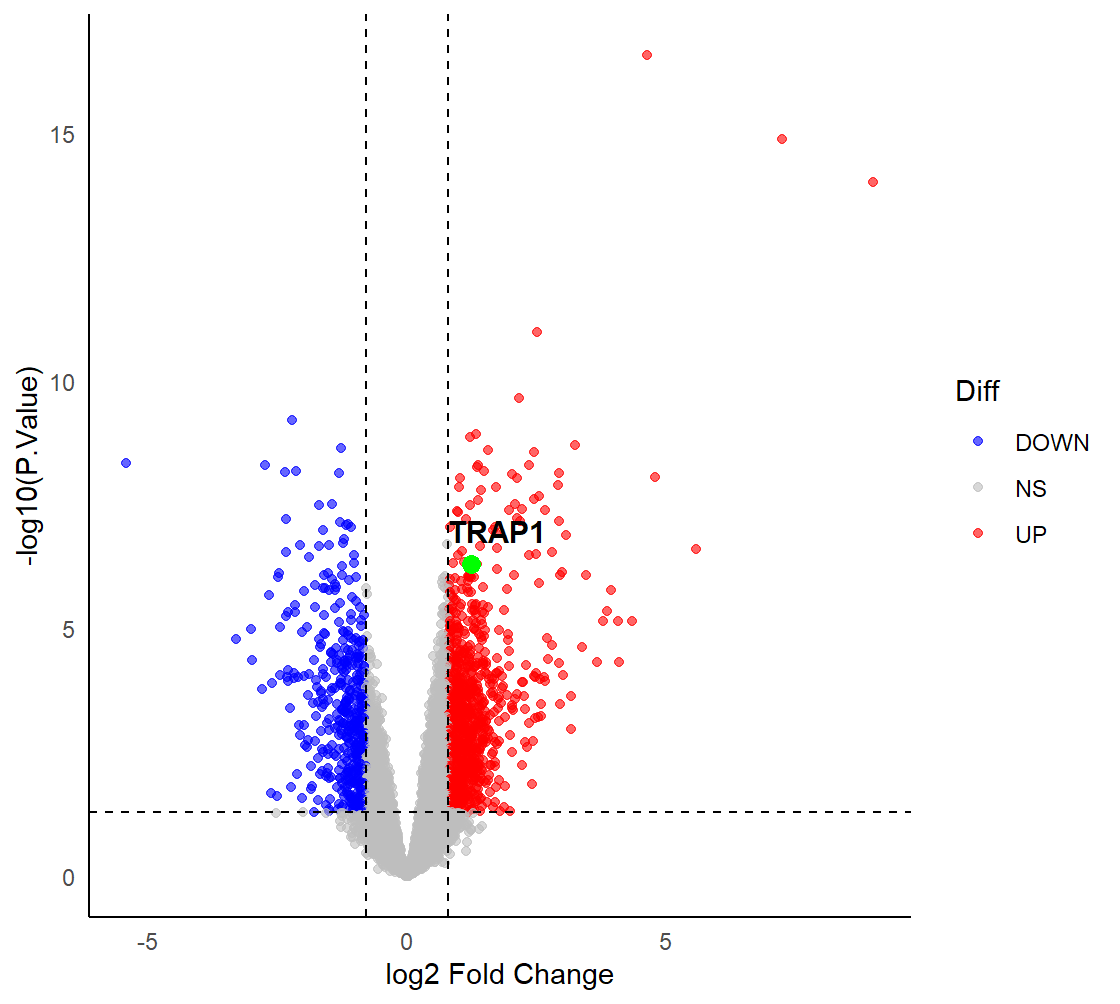


**Figure S2 The volcano plot shows that TRAP1 is upregulated in GC tissues.**


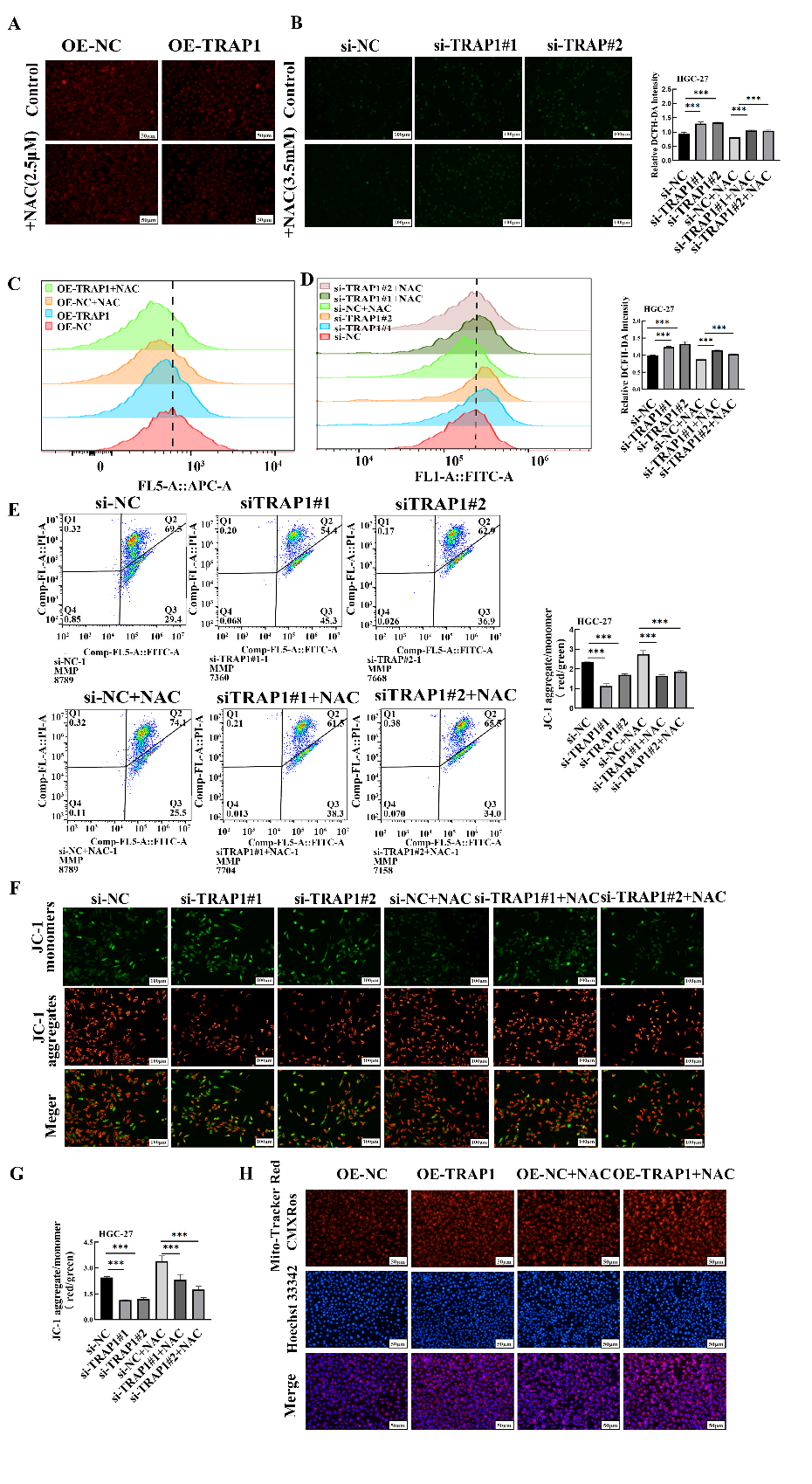


**Figure S3** **The combined action of NAC and TRAP1 can further inhibit the accumulation of ROS and maintain the MMP in GC cells.** (A-B) ROS levels in GC cells co-treated with TRAP1 overexpression and silencing and NAC, detected by fluorescent probe. (C-D) FCM analysis showed that NAC co-inhibited ROS accumulation with TRAP1 over-expressed, and NAC could reverse the increased ROS accumulation induced by TRAP1 silencing (E) NAC alleviates TRAP1 silencing-mediated MMP depolarization in HGC-27. cells, as analyzed by FCM. (F-G) MMP in GC cells co-treated with TRAP1 overexpression and silencing and NAC, detected by fluorescent probe. *: *P*<0.05; **: *P*<0.01; ***: *P*<0.001 vs control.


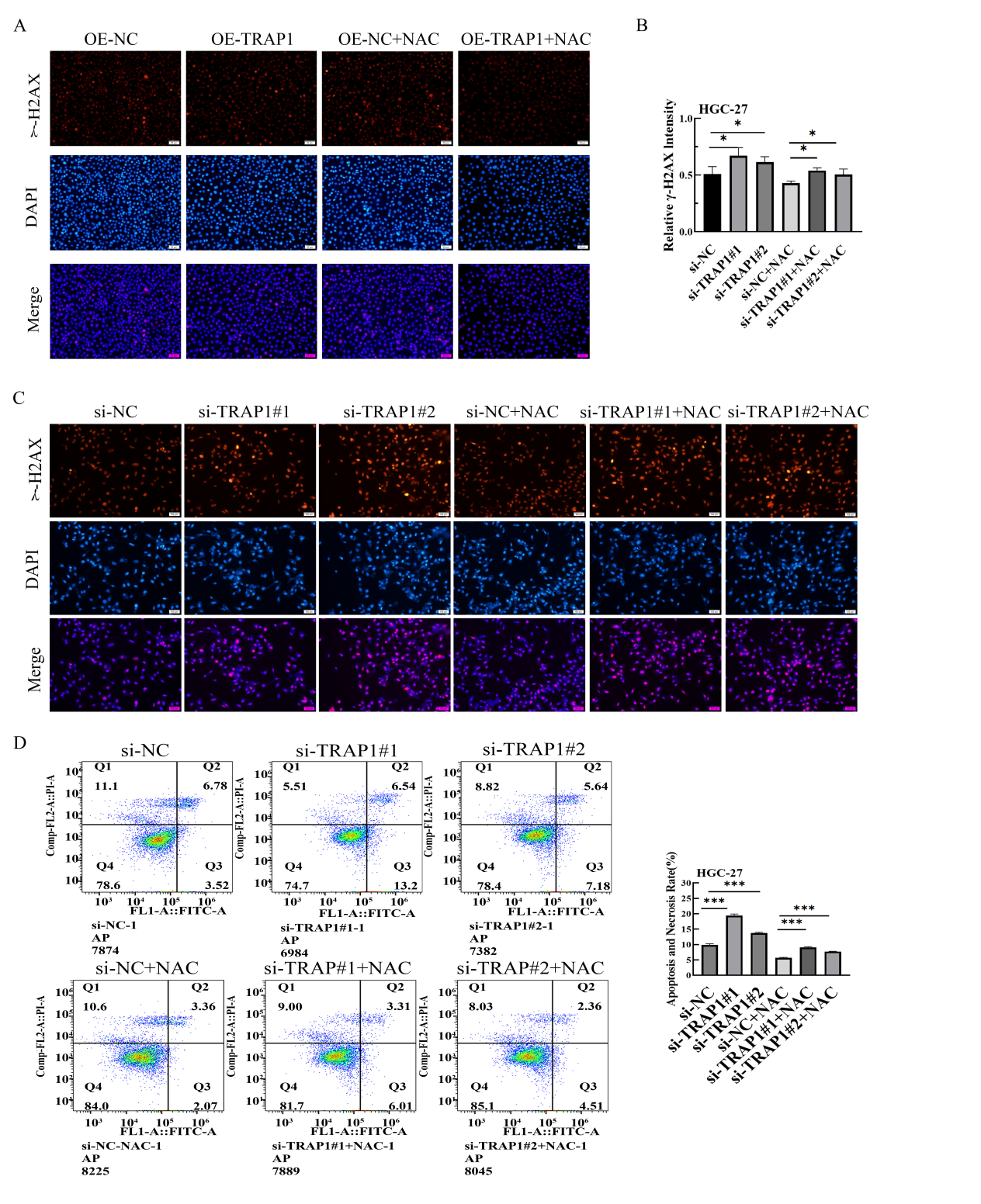


**Figure S4** **The combined action of TRAP1 and NAC can reduce DNA damage and reduces cell death in GC cells.** (A) NAC treatment reduced the expression of DNA damage marker γ-H2AX in TRAP1-overexpressing GC cells. (B-C) NAC treatment mitigated the TRAP1-silencing induced upregulation of γ-H2AX. (D) NAC alleviated TRAP1 silencing reduces cell death (including apoptosis and necrosis) in GC cells. *: *P*<0.05; **: *P*<0.01; ***: *P*<0.001 vs control.


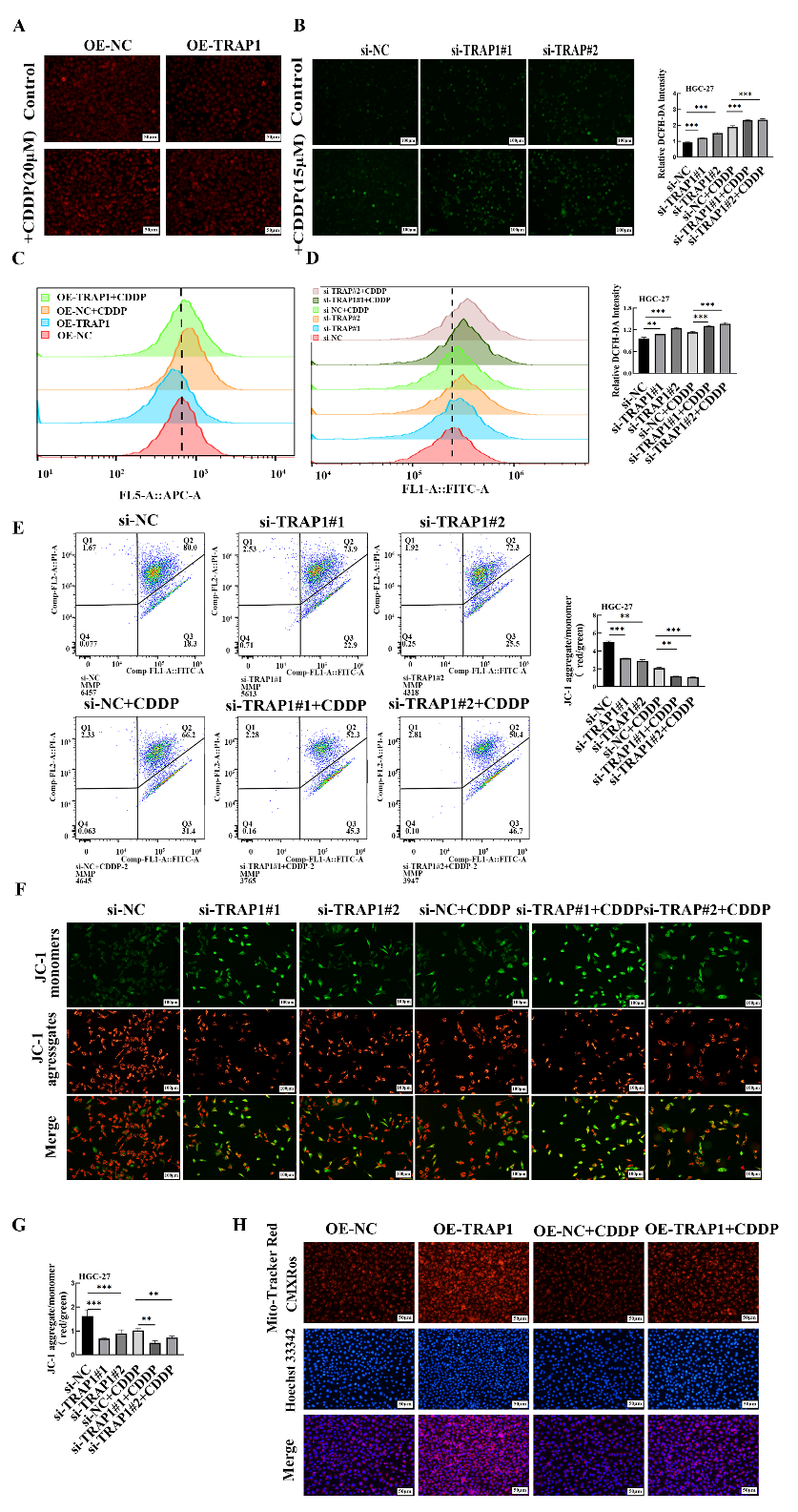


**Figure S5** **TRAP1 can reduce CDDP-induced accumulation of ROS and alleviate the decrease in MMP in GC cells.** (A-B) ROS levels in TRAP1-overexpressing and silenced GC cells treated with CDDP, detected by fluorescent probe. (C-D) FCM analysis showed that after CDDP treatment, TRAP1 overexpression inhibits ROS accumulation in GC cells, while TRAP1 silencing further increases ROS levels in GC cells. (E) FCM analysis confirmed that CDDP further decreased MMP levels in TRAP1-silenced HGC-27 cells. (F-G) MMP levels in TRAP1-overexpressing and silenced GC cells treated with CDDP, detected by fluorescent probe. *: *P*<0.05; **: *P*<0.01; ***: *P*<0.001 vs control.


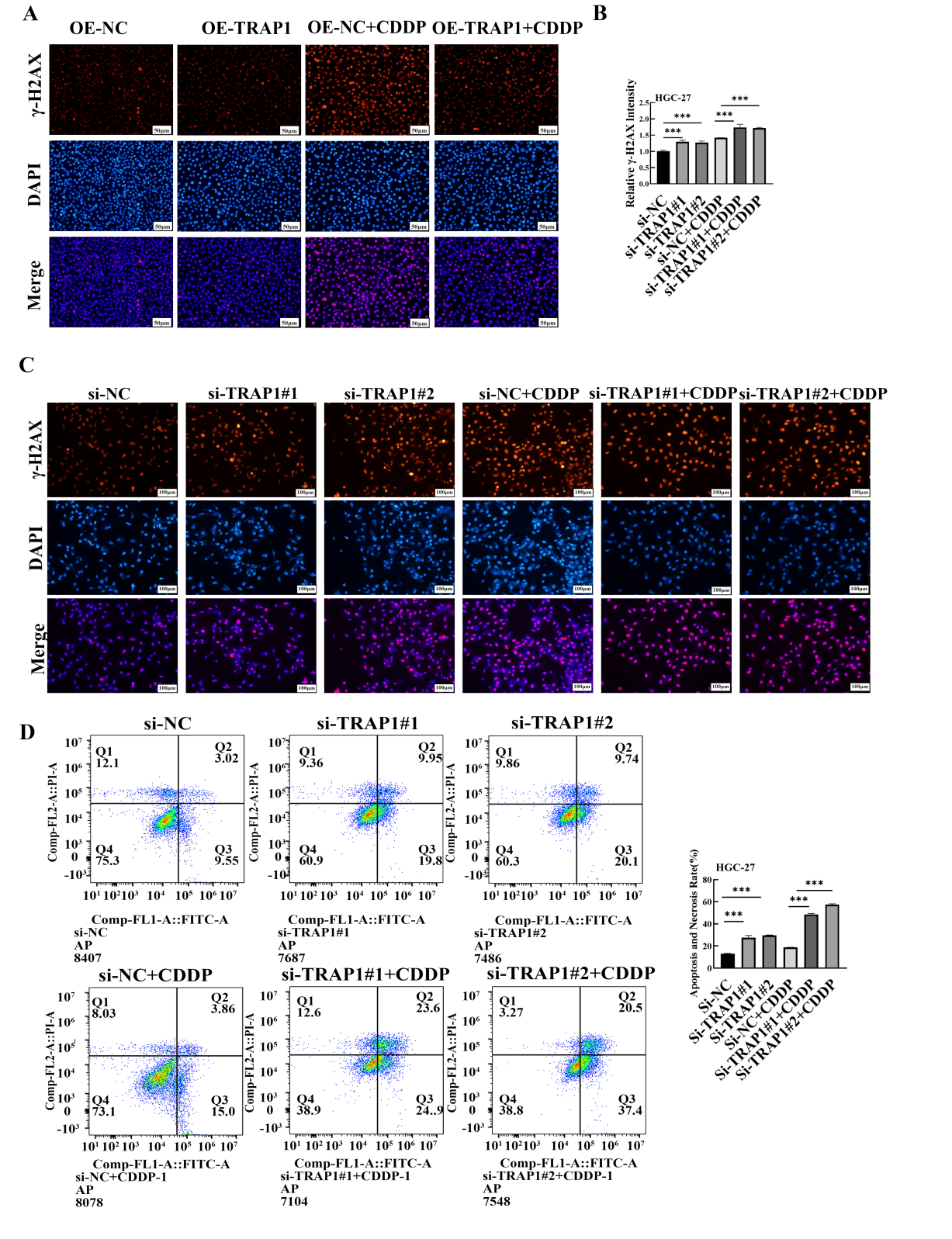


**Figure S6** **TRAP1 alleviates CDDP-induced DNA damage and** **reduces cell death in GC cells.** (A) Overexpression of TRAP1 reduces the CDDP-induced increase in γ-H2AX expression in GC cells. (B-C) CDDP treatment further promotes the increase in γ-H2AX expression in TRAP1 silencing GC cells. (D) CDDP treatment of TRAP1 silencing GC cells further increases cell death (including apoptosis and necrosis). *: *P*<0.05; **: *P*<0.01; ***: *P*<0.001 vs control.
